# Knee skin temperature following unicompartmental knee arthroplasty and its relationship with inflammatory indicators

**DOI:** 10.1002/jeo2.70593

**Published:** 2026-03-10

**Authors:** Dexin Lin, Xinchao Mao, Qi Zhuang, Weiqian Sun, Jing Wang, Gaoqu Zhang, Huachen Yu

**Affiliations:** ^1^ Department of Orthopedics The Second Affiliated Hospital and Yuying Children's Hospital of Wenzhou Medical University Wenzhou Zhejiang China

**Keywords:** infrared thermometer, knee skin temperature, serum inflammatory markers, temperature difference, unicompartmental knee arthroplasty

## Abstract

**Purpose:**

Unicompartmental knee arthroplasty (UKA) effectively alleviates pain and restores function in end‐stage knee osteoarthritis. However, the relationship between post‐operative knee skin temperature and systemic inflammatory responses remains poorly characterized. This study aimed to: (1) quantify changes in knee skin temperature and temperature difference for 6 months post‐UKA, (2) analyze correlations between temperature difference and serum inflammatory markers, (3) determine whether prolonged thermal alterations represent a normal healing response or potential early warning sign of complications.

**Methods:**

This study included 100 individuals who underwent UKA for primary osteoarthritis. Bilateral Knee skin temperatures were measured via infrared thermography preoperatively and at post‐operative days (PODs) 1, 3, 5 and months 1, 3, 6, with strict ambient temperature control (20 ± 1.0°C). Concurrently, erythrocyte sedimentation rate (ESR), white blood cell count (WBC) and serum C‐reactive protein (CRP) were assessed. Functional recovery was quantified using Hospital for Special Surgery (HSS) knee score.

**Results:**

A total of 100 patients participated in the study. Bilateral knee skin temperature and temperature difference peaked at POD 3 following UKA, with gradual normalization occurring over 6 months. The patient's CRP and WBC demonstrated progressive elevation until POD 3, while ESR exhibited delayed onset of increase. Subsequent measurements showed divergence in marker resolution: CRP and WBC levels initiate decline by POD 5, whereas ESR peaked at POD 5 following UKA. All inflammatory markers returned to preoperative levels during follow‐up.

**Conclusion:**

The skin temperature of the operated knee showed a rapid increase on the first POD following UKA, peaked on POD 3, and gradually returned to normal levels by 6 months after UKA. Moreover, there is a significant correlation between changes in temperature difference and serum inflammatory markers. Normal surgical reaction may cause this alteration.

**Level of Evidence:**

Level III.

**Abbreviations**
ASA: American Society of Anesthesiologists
BMI: body mass index
CRP: C‐reactive protein
ESR: erythrocyte sedimentation rate
HSS: Hospital for Special Surgery knee score
POD: post‐operative day
TKA: total knee arthroplasty
UKA: unicompartmental knee arthroplasty
WBC: white blood cell

## INTRODUCTION

Osteoarthritis of the knee has become the most prevalent joint disorder worldwide because of population growth and an ageing population, with radiographic evidence demonstrating a prevalence approaching 80% among individuals aged 65 years and older [2]. For patients with end‐stage unicompartmental disease, unicompartmental knee arthroplasty (UKA) has emerged as an effective surgical option, demonstrating implant survivability rates of 89.4% at 5 years and 80.6% at 10 years [12]. This bone‐ and ligament‐preserving procedure offers distinct advantages over total knee arthroplasty (TKA), including reduced hospitalization duration, lower post‐operative morbidity and superior patient satisfaction outcomes [20, 21]. The benefits of UKA extend beyond these immediate perioperative measures. Comparative studies have consistently shown that UKA results in shorter operative times, decreased blood loss, accelerated rehabilitation protocols and more physiological gait kinematics when compared with TKA [5, 9, 10, 13, 22].

However, clinical observations have identified a persistent skin temperature elevation around the operated knee following UKA. Many patients were concerned that post‐operative knee skin temperature increase may be related to infection. Obviously, to determine whether prolonged thermal alterations represent a normal healing response or infection is extremely important. While similar thermal patterns have been documented in TKA patients and are generally considered part of the normal post‐operative inflammatory response, no systematic investigation has examined this phenomenon specifically in the context of UKA [1]. This knowledge gap is particularly significant given the fundamental differences in surgical trauma and tissue preservation between UKA and TKA procedures. Therefore, we conducted a study on the changes in knee joint skin temperature and serum inflammatory indicators of patients after receiving UKA.

To address this unmet need, we conducted a retrospective study examining the temporal relationship between knee skin temperature changes and systemic inflammatory markers following UKA. This investigation aimed to: (1) characterize the natural history of post‐operative skin temperature changes after UKA, (2) correlate these changes with established inflammatory biomarkers, and (3) determine whether prolonged thermal alterations represent a normal healing response or potential early warning sign of complications.

## PATIENTS AND METHODS

### Patient population

Between January 2023 and January 2024, this retrospective study enroled patients who underwent unilateral UKA for primary osteoarthritis at The Second Affiliated Hospital of Wenzhou Medical University. The indications for UKA were anteromedial OA, intact knee ligaments, flexion contracture of <15°, preserved knee range of motion and a varus deformity of <15° that was correctable [8]. The patient inclusion criteria were: (i) men and women aged between 50 and 90 years; (ii) had no clinical evidence of urinary or pulmonary infection; and (iii) passive thermal measurements not following any physical activity or procedure other than UKA. Exclusion criteria are: (i) any infection within six months prior to surgery; (ii) serious underlying diseases that may affect body temperature and knee skin temperature (e.g., tumour, diabetes, cirrhosis, autoimmune diseases and hyperthyroidism); (iii) long‐term use of glucocorticoids; (iv) rheumatoid arthritis; and (v) unable to complete the periodic review.

This study was approved by our hospital's institutional review board (2025‐K‐309‐01), and it complied with the Declaration of Helsinki. Informed consent was obtained from all subjects involved in the study.

### Surgical technique and post‐operative treatment

All operations were performed by the same senior doctor with Oxford phase III (Biomet Ltd). The surgical approach was a minimally invasive medial parapatellar incision without patella dislocation. The incision was made from the medial pole of the patella to the medial side of the tibial tuberosity. The procedure followed a tibia‐first technique, with initial resection of the proximal tibia followed by preparation of the femoral condyle. Careful gap balancing was then performed to select the appropriate mobile bearing thickness. Finally, the cemented components were implanted, and the mobile bearing was inserted. The wound was closed in layers following standard protocols. Post‐operatively, a standardized protocol was followed for recovery. Intravenous antibiotics were administered prophylactically for 24 hours to prevent surgical site infection. Rivaroxaban was prescribed for two weeks to minimize the risk of venous thromboembolism. A structured rehabilitation programme, emphasizing early mobilization, range of motion exercises, and muscle strengthening, was initiated under the supervision of a physiotherapist on the first post‐operative day (POD).

### Study methodology

We documented each participant's baseline demographics—sex, age, height, weight and body mass index (BMI)—along with their American Society of Anesthesiologists (ASA) physical status classification (Grade 1 = *healthy*/Grade 5 = *moribund*) [7]. Preoperative radiographic severity of osteoarthritis in both knees was determined utilizing Kellgren–Lawrence (K‐L) scale (Grade 0 = *normal*/Grade 4 = *severe*) [4]. Functional performance of the operated knee was evaluated by the Hospital for Special Surgery (HSS) knee score (0–100) before surgery and at 6 months post‐operatively [18].

A calibrated handheld infrared thermography device (Hikvision) was utilized to record skin temperatures on bilateral knees. The instrument demonstrated high metrological precision, measuring from −20°C to 100°C, with ±0.4°C accuracy and 0.2°C repeatability. The thermal imager featured rapid response characteristics (300 ms acquisition time) and maintained measurement stability across operational environmental conditions (including temperatures from −10°C to 50°C and relative humidity from 10% to 95%) [23].

**Figure 1:**
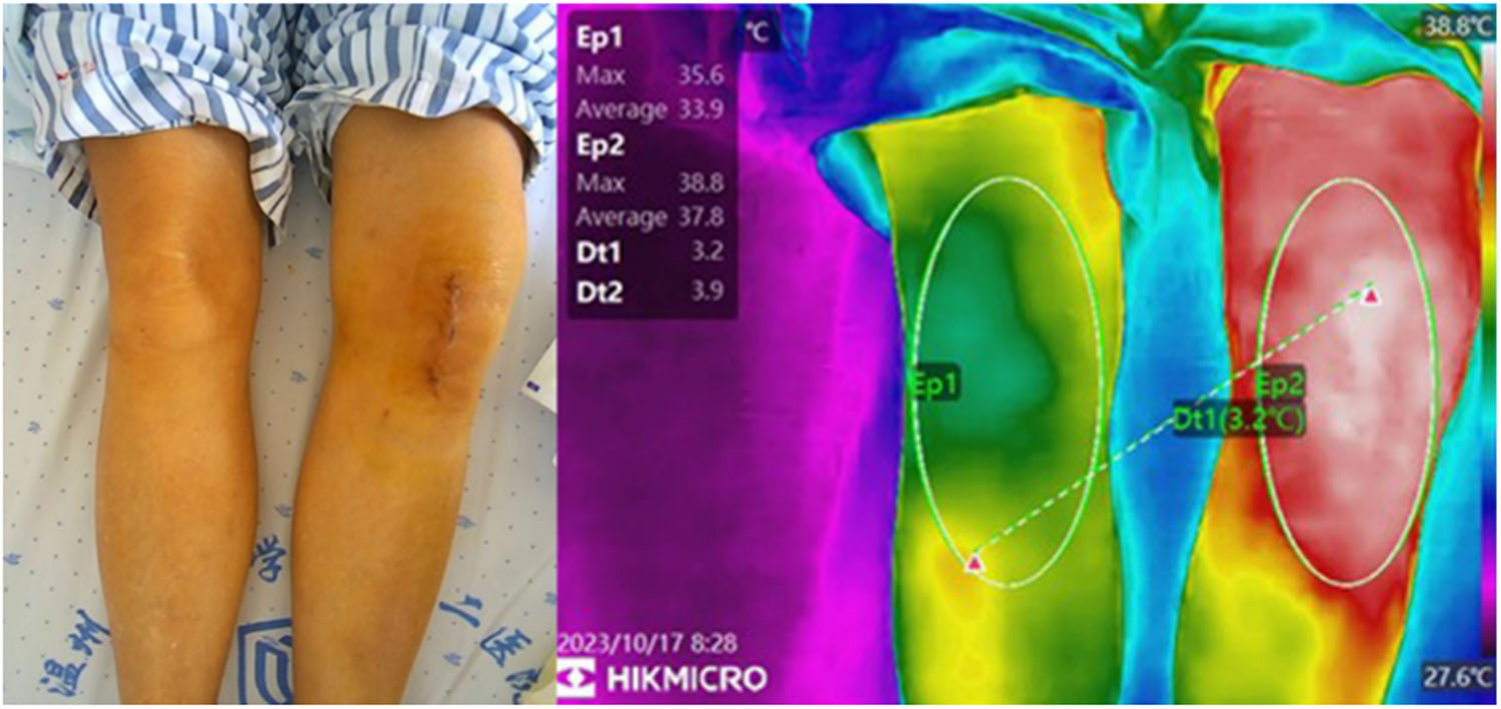
This representative patient underwent UKA 1 week post‐operatively. For the skin knee temperature measurements, patients were lying flat with both knees extended in a controlled environment of 20.0 ± 1.0°C and 50.0 ± 10.0% humidity. Skin temperature was measured using an infrared thermometer, and this patient showed a 3.2°C temperature difference between the operated and contralateral knees. UKA, unicompartmental knee arthroplasty.

Bilateral knee skin temperature was measured by a trained investigator at the following time points: 1 day preoperatively and PODs 1, 3 and 5, followed by months 1, 3 and 6. All measurements were conducted in a climate‐controlled environment (20.0 ± 1.0°C; 50.0 ± 10.0% relative humidity) between 09:00–12:00 and 14:00–17:00 hours. Participants maintained a supine position with knees fully extended, having exposed both lower limbs for 15–20 min of thermal equilibration prior to measurement. Infrared thermographic assessment was performed with the device positioned at a standardized 1‐m distance from the target anatomical sites (Figure 1). The average temperature within the circle is used as the approximate temperature within the knee skin temperature. Knee skin temperature differentials were classified as follows: mild elevation (<1.0°C), moderate elevation (1.0–3.0°C) or severe (>3.0°C) [15].

At each study time point, 3 mL of venous blood was drawn in sterile tubes and maintained at ambient temperature until analysis. Preoperative and post‐operative serum inflammatory markers (erythrocyte sedimentation rate [ESR], C‐reactive protein [CRP] and white blood cell [WBC]) were quantified using standardized biochemical assays.

### Statistical analysis

Although no formal sample size calculation was performed, this study included 100 eligible patients. A repeated‐measures analysis of variance (ANOVA) was used to compare the temperature of both knees at each clinic visit. Differential temperatures across visits and HSS scores were analysed using one‐way analysis of variance. The relationship between the variables was assessed using Pearson's correlation coefficient. Selected multivariate linear regression model to evaluate the relationship between the variables.

All statistical procedures utilized SPSS® 25.0, declaring *p* < 0.05 as statistically significant.

## RESULTS

The cohort included 100 patients (34 men and 66 women) with a mean age of 69.3 ± 8.0 years (range: 54–88 years). Anthropometric measurements revealed a mean height of 160.4 ± 6.7 cm (range: 145–176 cm) and a mean weight of 67.5 ± 10.5 kg (range: 45.0–103.0 kg). All surgical knees demonstrated K–L Grade 4 radiographic osteoarthritis. Post‐operative recovery was uneventful in all cases, with no patients developing progressive contralateral knee symptoms during the 6‐month follow‐up period.

**Figure 2:**
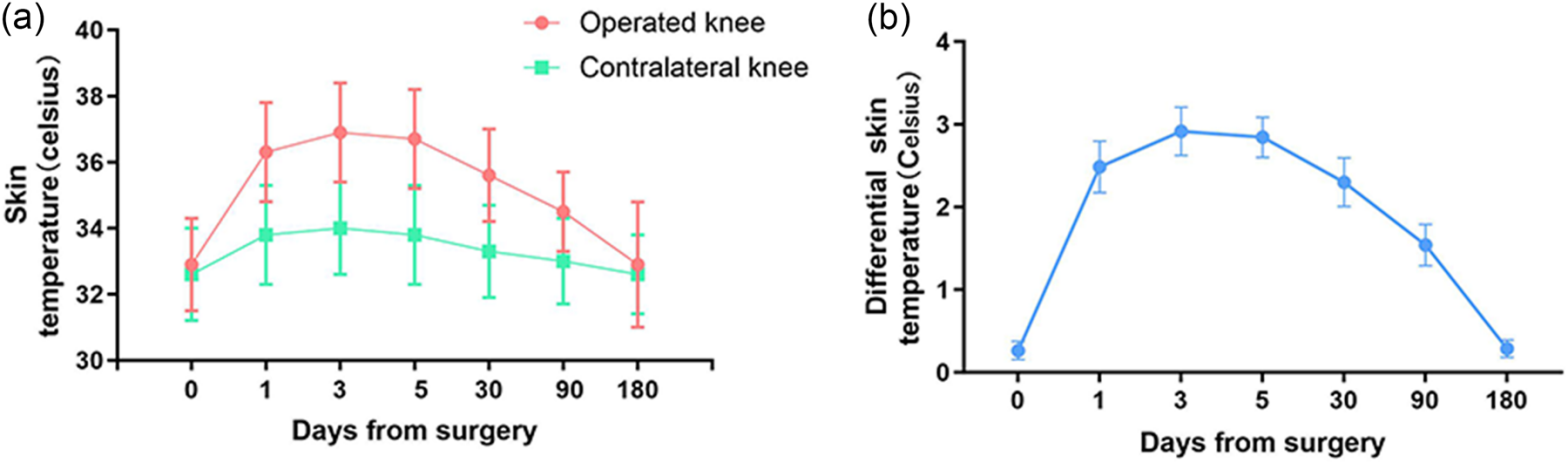
Skin temperature changes for the operated and contralateral knees (a) and the differential between the two knees (b) before and for 6 months following unicompartmental knee arthroplasty (*n* = 100).

Table 1 summarizes pre‐ and post‐operative skin temperatures of the operated knee, temperature differentials and serum indices. After surgery, the operated knee's surface temperature climbed, reaching its apex on Day 3. Repeated measures ANOVA showed that, with the exception of the preoperative visit, the differences between the skin temperatures of both knees were statistically significant at each visit (*p* < 0.001) (Table 2). While the contralateral knee regained near‐baseline values by Day 30, the operated knee required six months to normalize (Figure 2a). By the way, Day 3 showed the most pronounced differential knee skin temperature (Figure 2b).

**Table 1:** Values of knee skin temperature and serum indices before and for 6 months following unicompartmental knee arthroplasty (*n* = 100).

Independent variable	Post‐operative time points						
	Preop	1 day	3 days	5 days	30 days	90 days	180 days
Operated knee, °C	32.9 ± 1.4	36.3 ± 1.5	36.9 ± 1.5	36.7 ± 1.5	35.6 ± 1.4	34.5 ± 1.2	32.9 ± 1.9
Contralateral knee, °C	32.6 ± 1.4	33.8 ± 1.5	34.0 ± 1.4	33.8 ± 1.5	33.3 ± 1.4	33.0 ± 1.3	32.6 ± 1.2
Differential, °C	0.3 ± 0.1	2.5 ± 0.3	2.9 ± 0.3	2.8 ± 0.2	2.3 ± 0.3	1.5 ± 0.3	0.3 ± 0.1
WBC, 10^9^/L	7.1 ± 2.0	9.9 ± 2.5	10.5 ± 2.4	8.6 ± 1.7	7.0 ± 1.5	6.9 ± 1.4	6.9 ± 1.1
ESR, mm/h	13.4 ± 6.3	18.3 ± 9.8	24.3 ± 10.6	30.7 ± 8.7	16.7 ± 3.9	12.6 ± 5.9	12.1 ± 5.4
CRP, mg/dL	3.6 ± 2.9	16.1 ± 12.0	39.0 ± 31.0	21.6 ± 13.0	4.3 ± 2.1	3.5 ± 1.3	3.2 ± 0.9

*Note*: Values are shown as mean ± SD. The knee skin temperature was taken as a mean value of four areas (superolateral, superomedial, inferiorlateral and inferiormedial borders of the patella).

Abbreviations: CRP, C‐reactive protein; ESR, erythrocyte sedimentation rate; Preop, preoperative time point; SD, standard deviation; WBC, white blood cell.

**Table 2:** A repeated‐measures ANOVA of the changes in knee skin temperature in patients who had undergone unicompartmental knee arthroplasty (*n* = 100).

	Post‐operative time points						
	Preop	1 day	3 days	5 days	30 days	90 days	180 days
Operated knee, °C	32.9 ± 1.4	36.3 ± 1.5	36.9 ± 1.5	36.7 ± 1.5	35.6 ± 1.4	34.5 ± 1.2	32.9 ± 1.9
Contralateral knee, °C	32.6 ± 1.4	33.8 ± 1.5	34.0 ± 1.4	33.8 ± 1.5	33.3 ± 1.4	33.0 ± 1.3	32.6 ± 1.2
Group effects	*F* = 484.1; *p* < 0.001						
Between‐group effects	*F* = 119.0; *p* < 0.001						
Group × Between‐group	*F* = 89.7; *p* < 0.001						

Abbreviation: ANOVA, analysis of variance.

**Figure 3:**
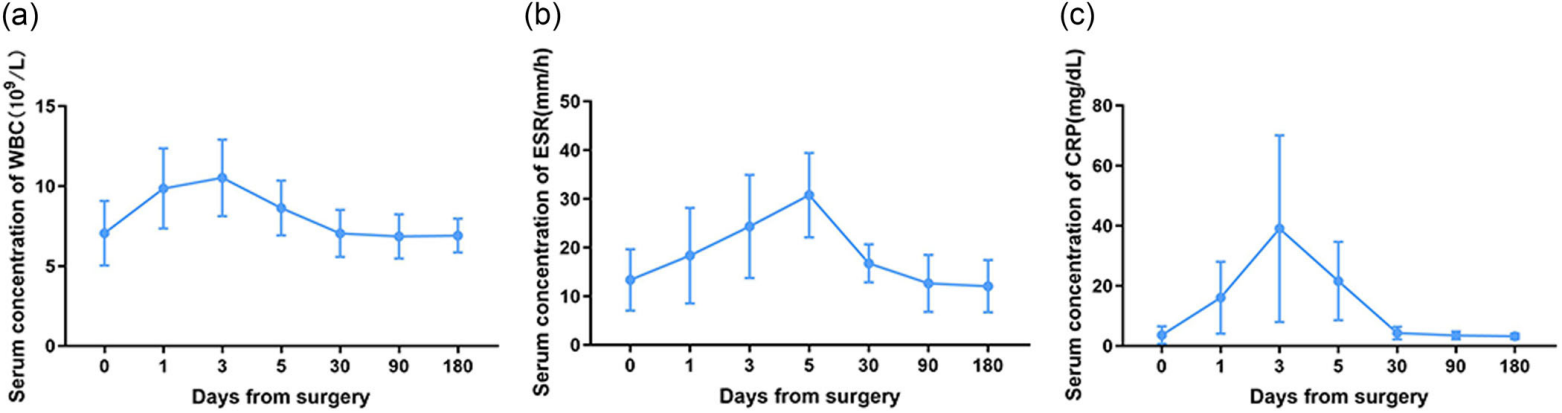
Serum indices taken preoperatively and for up to 6 months post‐operatively following UKA (*n* = 100): (a) WBC, white blood cells; (b) ESR, erythrocyte sedimentation rate; (c) CRP, C‐reactive protein. UKA, unicompartmental knee arthroplasty.

WBC levels and CRP proteins reached their maxima on POD 3, returning to baseline by Day 30 (Figure 3a,c). ESR peaked on Day 5 and normalised by Day 90 (Figure 3b). Operated‐knee skin temperature was significantly positively linked to WBC, ESR and CRP and negatively correlated with days from surgery (*p* < 0.05). WBC, ESR and CRP were also significantly positively associated with differential temperature and negatively related to days from surgery (*p* < 0.05) (Table 3).

**Table 3:** Pearson's correlation coefficient analysis of the possible correlation between knee skin temperature and other parameters in patients who had undergone unicompartmental knee arthroplasty (*n* = 100).

Independent variable		Operated knee temperature			Contralateral knee temperature		Differential temperature
	*r*	Statistical significance		*r*	Statistical significance	*r*	Statistical significance
K–L classification	0.132	NS		0.194	NS	0.146	NS
Age, years	−0.099	NS		−0.103	NS	0.035	NS
Height, m	0.110	NS		0.112	NS	0.088	NS
Weight, kg	0.149	NS		0.166	NS	0.186	NS
BMI, kg/m^2^	0.122	NS		0.140	NS	0.215	NS
ASA Score	0.146	NS		0.234	NS	0.269	NS
WBC, 10^9^/L	0.775	*p*＜0.05		0.870	*p*＜0.05	0.722	*p*＜0.05
ESR, mm/h	0.813	*p*＜0.05		0.817	*p*＜0.05	0.729	*p*＜0.05
CRP, mg/dL	0.773	*p*＜0.05		0.844	*p*＜0.05	0.786	*p*＜0.05
POD	−0.386	*p*＜0.05		−0.388	*p*＜0.05	−0.205	*p*＜0.05

Abbreviations: BMI, body mass index; CRP, C‐reactive protein; ESR, erythrocyte sedimentation rate; K–L, Kellgren–Lawrence; NS, not statistically significant (*p* ≥ 0.05); POD, post‐operative day; r, Pearson's correlation coefficient; WBC, white blood cell.

Multivariable regression analysis using selective variables from the simple regression found that for the operated knee skin temperature, BMI, WBC, ESR and CRP showed positive correlations (*p* < 0.05), while age and days from surgery showed an inverse correlation (*p* < 0.05). For the contralateral knee skin temperature, BMI showed a positive correlation (*p* < 0.05), and age and days from surgery showed an inverse correlation (*p* < 0.05). For the differential temperature change, days from surgery showed inverse correlations (*p* < 0.05), and BMI and WBC showed positive correlations (*p* < 0.05) (Table 4).

**Table 4:** Multivariable regression analysis of independent predictors of changes in knee skin temperature in patients who had undergone unicompartmental knee arthroplasty (*n* = 100).

	Operated knee temperature		Contralateral knee temperature		Differential temperature		
Independent variable	*β*	Statistical significance	*β*	Statistical significance	*β*		Statistical significance
K–L classification	Excluded		Excluded		Excluded		
Age, years	−0.015	*p* < 0.05	−0.037	*p *< 0.05	−0.004		NS
Height, m	Excluded		Excluded		Excluded		
Weight, kg	Excluded		Excluded		Excluded		
BMI, kg/m^2^	0.013	*p* < 0.05	0.011	*p *< 0.05	0.014		*p* < 0.05
ASA Score	Excluded		Excluded		Excluded		
WBC, 10^9^/L	0.156	*p* < 0.05	Excluded		0.245		*p* < 0.05
ESR, mm/h	0.324	*p* < 0.05	0.017	NS	0.022		NS
CRP, mg/dL	0.336	*p* < 0.05	0.012	NS	0.031		NS
POD	−0.439	*p* < 0.05	−0.267	*p *< 0.05	−0.213		*p* < 0.05

Abbreviations: ASA, American Society of Anesthesiologists; BMI, body mass index; CRP, C‐reactive protein; ESR, erythrocyte sedimentation rate; K–L, Kellgren–Lawrence; NS, not statistically significant (*p* ≥ 0.05); POD, post‐operative day; WBC, white blood cell.

Table 5 lists baseline and final HSS knee scores for participants stratified by mild, moderate and severe inter‐knee temperature elevations. One‐way ANOVA showed that the mild subgroup achieved a markedly larger mean ± standard deviation improvement between the preoperative and last follow‐up visits than the moderate (*p* < 0.05) or severe (*p* < 0.05) subgroups. Pearson's correlation coefficient analysis showed an inverse correlation between the grade of differential temperature elevation and the HSS score improvement (*r* = 0.75, *p* < 0.05).

**Table 5:** Hospital for Special Surgery (HSS) knee scores and differential knee temperatures for patients who had undergone unicompartmental knee arthroplasty (*n* = 100).

Factors	Elevation in differential temperature		
	Mild (*n* = 29)	Moderate (*n* = 53)	Severe (*n* = 18)
Differential skin temperature, °C	0.42 ± 0.21	2.13 ± 0.74	3.66 ± 0.53
HSS score			
Preoperative visit	54.27 ± 2.67	56.34 ± 1.84	58.63 ± 1.76
Last follow‐up visit	89.61 ± 2.97	86.51 ± 2.03	88.43 ± 2.58
Difference between visits	38.33 ± 4.51	33.85 ± 1.67	34.73 ± 2.45

*Note*: Values are shown as mean ± SD. The differential temperature (i.e., operated knee minus the contralateral knee) was categorized as a mild (<1.0°C), moderate (1.0–3.0°C) or severe elevation (>3.0°C). *p* < 0.05 compared with moderate or severe elevation groups.

Abbreviation: SD, standard deviation.

## DISCUSSION

This investigation offers the first systematic portrayal of bilateral knee thermography, temperature disparities and serum inflammatory profiles across the 6 months after UKA. A pronounced increase in temperature differential appears during the first three PODs, subsides gradually to baseline by one month, and remains comparable to preoperative readings at 6 months, a time point at which patients generally exhibit satisfactory, complication‐free clinical outcomes.

Maintaining a constant ambient temperature is essential when capturing skin‐temperature data. Despite the potential fluctuation caused by morning‐to‐afternoon changes in blood flow and heat distribution, the infrared thermographic device used here provided reliable values [6, 14]. Knee thermography constitutes a reproducible, safe and non‐invasive approach for identifying joint activity in inflammatory arthritis. Follow‐up scans can be performed swiftly during outpatient appointments. This technique accurately detects subtle changes in local blood flow and microcirculation, serving as an objective tool to monitor inflammation and tissue healing.

This research has found that, prior to surgery, osteoarthritic knees exhibited a greater temperature gap relative to the unaffected limb. Gavish et al. similarly documented a preoperative mean differential of 0.3 ± 0.1°C, approaching the 0.5°C threshold considered clinically significant in symmetric limbs. This baseline disparity likely reflects the pathophysiological changes inherent to knee osteoarthritis in most patients [3]. Conversely, those with bilateral osteoarthritis or other symmetric arthritic diseases, as rheumatoid arthritis, may not exhibit a significant temperature gap [19]. The study analysis demonstrated that both the operated and contralateral knees experienced elevated skin temperatures—and their differential—following UKA. The observed skin temperature elevation during the early post‐operative period following UKA was expected due to acute inflammatory responses and surgical trauma. As healing progresses, the inflammatory response decreases, leading to a gradual normalization of temperature differences. However, temperature differentials of at least 0.5°C remained for 1 month, potentially indicating ongoing remodelling of patellar blood flow or skin microcirculation in the surgical area [16]. By the 6‐month evaluation, skin temperatures had reverted to their preoperative levels, indicative of successful clinical recovery. The findings provide a new quantitative indicator for post‐operative recovery and valuable insights into the local inflammatory response.

In addition to temperature changes, systemic inflammatory markers in this study were evaluated: CRP: Peaks early after surgery, mirroring the acute inflammatory response and declines as healing occurs. ESR: Remains elevated longer than CRP, which may explain the persistent temperature differences observed between 1 and 3 months. WBC: Shows a transient response to surgical trauma and is less sensitive to localized inflammation, resulting in a weaker correlation with temperature changes. Kılıçarslan et al. reported that the UKA patients had significantly higher ESR and CRP levels on the 3rd and the 15th POD, while the ESR and CRP levels normalized after the first month [11]. The result is similar to this study. However, this study first report that both the operated knee's surface temperature and the inter‐knee thermal gap are highly positively associated with circulating WBC, ESR and CRP. These observations indicate that CRP and ESR are more closely associated with the inflammatory processes affecting knee temperature and can complement thermographic assessments in monitoring recovery. This means that changes in serum inflammatory markers are likely a normal response after surgery.

The study found that UKA patients exhibited nearly normal bilateral knee temperature symmetry by 6 months post‐operatively, which is much shorter than TKA skin temperature normalization. Zeng et al evaluated 39 post‐TKA patients and reported that elevated skin temperature may represent a normal surgical response lasting up to 12 months post‐operatively [24]. In a larger series exceeding 1000 patients, Sharma et al. demonstrated a significant rise in skin temperature extending to 12 months post‐TKA [17]. This difference may be attributed to the less invasive nature and shorter operative time of UKA, leading to a milder inflammatory response and quicker restoration of local microcirculation.

There are several limitations to consider. First, the limited number of enroled patients reduces the robustness of the conclusions, warranting follow‐up studies with larger cohorts. Second, the overly female‐skewed sample may limit how widely these findings can be generalized. Eventually, intraoperative factors such as implant size and tourniquet duration were not evaluated, and no cases of joint infection were observed, which prevented analysis of temperature changes in relation to periprosthetic joint infection.

## CONCLUSION

This study is the first to elucidate the dynamic changes in knee temperature, temperature difference and serum inflammatory markers up to six months in uncomplicated UKA patients. Further investigations of changes in operated knee temperature in patients at different time points post‐UKA and correlative serum inflammatory markers, demonstrating that post‐operative bilateral knee temperature and inflammatory markers normalize by six months may be a normal surgical response.

## AUTHOR CONTRIBUTIONS

Dexin Lin, Xichao Mao, Qi Zhuang and Huachen Yu conceived and designed the study. Dexin Lin, Weiqian Sun, Jing Wang and Huachen Yu supervised the conduct of the study. Gaoqu Zhang analysed the data. Dexin Lin wrote the initial drafts. All authors critically revised the manuscript and ensured the accuracy of the data and analysis. All authors have seen and agree with the contents of the manuscript and agree that the work has not been submitted or published elsewhere in whole or in part.

## CONFLICT OF INTEREST STATEMENT

The authors declare no conflicts of interest.

## ETHICS STATEMENT

All procedures adhered to the ethical standards of the institutional and national research committees, the 1964 Declaration of Helsinki, and subsequent amendments. The study was approved by our hospital's institutional review board (2025‐K‐309‐01). Informed consent was obtained from all subjects involved in the study. Participants in this study were informed of and provided consent for the collection and use of their data.

## Data Availability

The data that support the findings of this study are available from the corresponding author upon reasonable request.
